# Are You Awed Yet? How Virtual Reality Gives Us Awe and Goose Bumps

**DOI:** 10.3389/fpsyg.2018.02158

**Published:** 2018-11-09

**Authors:** Denise Quesnel, Bernhard E. Riecke

**Affiliations:** iSpaceLab, School of Interactive Arts and Technology, Simon Fraser University, Surrey, BC, Canada

**Keywords:** positive technology, virtual reality, immersive technologies, emotions, emotion induction, awe, experience design

## Abstract

“Awe” is a category of emotion within the spectrum of self-transcendent experiences. Awe has wellness benefits, with feelings of social interconnectivity and increased life satisfaction. However, awe experiences remain rare in our everyday lives, and rarer in lab environments. We posit that Virtual Reality (VR) may help to make self-transcendent and potentially transformative experiences of awe more accessible to individuals. Here, we investigated how interactive VR as a positive technology may elicit awe, and how features of aesthetic beauty/scale, familiarity, and personalization (self-selection of travel destinations) may induce awe. In this mixed-methods study, participants used an interactive VR system to explore Earth from ground and orbit. We collected: introspective interviews and self-report questionnaires with participants’ experience of awe; information on personality traits and gender; and we recorded physiological goose bumps on the skin (using an arm-mounted goose bump camera instrument), which is a documented marker of an awe experience. Results showed that on a scale of 0–100 for self-reported awe, four different interactive VR environments yielded an average awe rating of 79.7, indicating that interactive VR can indeed induce awe. 43.8% of participants experienced goose bumps: awe ratings positively correlated with the occurrence of goose bumps with those who experienced goose bumps having showed significantly higher ratings of awe than those who did not. Most (64%) of the goose bumps occurred when participants self-selected their VR environment. Participant statements from the interviews were characteristic of an awe-inspiring experience, revealed themes of social connection, and usability problems with the VR interface. Personality traits yielded no clear correlation to awe ratings, and females appear to experience more goose bumps than males. In summary: (1) Interactive VR can elicit awe, especially within familiar, self-selected environments; (2) Physiological goose bumps can be recorded to provide reliable, non-intrusive indications of awe; (3) Care must be taken to design interaction interfaces that do not impede awe; and (4) While personality traits are not correlated to awe ratings, goose bumps were experienced more frequently among females. We aim to conduct future studies using custom VR environments, interfaces, and additional physiological measures to provide further insight into awe.

## Introduction

A transformative experience is an event in which an individual’s worldview is reconstructed, resulting in a shift in perspective or change to an individual’s identity (C’de Baca and Wilbourne, 2004; Piff et al., 2015; Gaggioli, 2016). These changes can be positive, and transformative experiences tend to support a long-term change that comes with this worldview reconstruction (Gaggioli, 2016). Many people who have experienced such changes reported feeling a eureka, “a-ha,” or epiphany-like moment as a “peak experience” (Miller and C’de Baca, 2011).

To understand a transformative experience, researchers search for emotions to measure, leading to “awe” identified as an emotion of interest. Specifically, awe is a positive emotion with self-transcendence qualities (Prade and Saroglou, 2016; Yaden et al., 2017; Chirico and Yaden, 2018), in which an individual feels connected to the universe and others. Awe has two core features, “**perceived vastness**,” and “**a need for accommodation**” (Keltner and Haidt, 2003). Individuals in “awe” often do not fully understand their experience in the moment and will make changes to their mental model to comprehend the scale of the situation afterward (Stepanova et al., under review). Keltner and Haidt (2003) and Schneider (2009) stress the potential of awe-inspiring experiences to be among the most powerful personal transformational experiences. Awe-elicited shifts feel pleasant, with focus transcending from the self to the needs of many (Stellar et al., 2017b). Experiences of awe can be very positive, characterized by social interconnectivity (Shiota et al., 2007; Schurtz et al., 2011; Prade and Saroglou, 2016), pro-sociality (Piff et al., 2015; Stellar et al., 2017b), and increased well-being and life satisfaction (Rudd et al., 2011; Cappellen et al., 2013; Stellar et al., 2015). Many go to lengths to seek out awe-inspiring experiences, especially those that lead to a sense of oneness with others (Van Cappellen and Saroglou, 2012); examples include musical concerts, spiritual retreats, and travel to monuments. With awe being such a profound experience, researchers are increasingly interested in learning how to elicit and study it in detail.

One of the most intense awe-inspiring experiences may be the sight of the Earth from Space, coined the “Overview Effect” (White, 2014). Research by Yaden et al. (2016) explored astronaut accounts of the Overview Effect, demonstrating awe and self-transcendence. We can see an example through astronaut Kathryn D. Sullivan:

“It’s hard to explain how amazing and magical this experience is… If you float up by the forward seats, you have six large windows providing you with a spectacular panorama of the Earth below, spanning at least 180 degrees of the horizon. I’m happy to report that no amount of prior study or training can fully prepare anybody for the awe and wonder this inspires.” (Sullivan, 1991).

However, most will never get to see the Earth from space, but astronaut accounts provide us with a typology of awe to learn from (Stepanova et al., under review). Awe is also elicited by being in nature, through stargazing, witnessing solar eclipses and beautiful vistas, seeing art and architecture (Keltner and Haidt, 2003; Shiota et al., 2007; Grewe et al., 2011; Gordon et al., 2017); even technology, like social media with its vastness of data and ability to connect people, can be awe-inspiring (Bai et al., 2017). Yet, experiences that elicit awe can be quite rare. How do we gain awe and personal, positive transformation in our lives? For many individuals with limitations to income, mobility, and ability, these awe experiences listed above are inaccessible and are difficult to integrate into daily lives. Thus, we look at new ways in which technology can create a new category of awe experiences.

## Virtual Reality as a Potential Solution for Eliciting Awe

### The Properties of Virtual Reality

A potential solution to accessibility of awe-inspiring experiences is immersive Virtual Reality (VR), a technological medium (Chirico et al., 2017; Quesnel and Riecke, 2017; Chirico et al., 2018). VR consists of a computer-generated immersive virtual environment, where the user may interactively act upon the environment and objects within it. Well-designed VR can help an individual become immersed in what feels like a believable experience akin to reality. Places and experiences that would be otherwise impossible, are made to feel possible, like time travel (Friedman et al., 2014) and visual reorientation illusions experienced by astronauts (Mast and Oman, 2004). There are core features that allow for this; immersion, interactivity, and presence, the latter a sense of “being there” in a virtual environment. Immersion is a sense of existing in a virtual environment, due to vividness and a sense of depth that a 3D environment enables, along with a breadth of provided information through multisensory cues, such as image, audio, olfactory signals, and tactile sensations (Ryan, 1999). With added interactivity, an individual can modify their environment so their actions have consequences, with the degree of interactivity being variable (Steuer, 1992). Immersion and interactivity alone are not responsible for subjective presence, as research has demonstrated that affective VR content influencing emotional intensity has an effect on levels of presence felt and adds relevance to the experience (Baños et al., 2004; Riva et al., 2007). While VR as a technological environment itself may not lead to elicitation of specific emotional states, Diemer et al. (2015) provide evidence that when participants feel emotionally affected, the overall level of presence increases. This can be achieved through the use of narrative and aesthetics (Bouchard et al., 2008; Gorini et al., 2011; Felnhofer et al., 2015; Triberti and Riva, 2016). Studies tend to focus on the general change in emotional state of a participant, opposed to elicitation of a specific emotion like awe, so more research is needed in exploring how VR features affect emotional specificity. This current study was designed to be a step toward addressing this gap.

### Research on Virtually Induced Awe

In psychology research, VR has advantages over other commonly used media stimuli: (1) environment can be personalized for the participant; (2) experimental control over the stimulus can be maintained; (3) high naturalism/believability due to its being a multimodal sensory device, which can lead to realistic behaviors (Wilson and Soranzo, 2015). Previous studies have explored the role of immersive video, virtual and mixed reality in eliciting awe. These include a mixed-reality Cave Automatic Virtual Environment (CAVE) by Gallagher et al. (2015), designed to elicit the Overview Effect; immersive 360 degree videos were used by Chirico et al. (2017); and interactive virtual environments with a VR headset (Chirico et al., 2018). These studies used surveys for assessing awe, with participants rating high levels of awe. Researchers have also used VR to investigate participant’s perceptions: Rauhoeft et al. (2015) used a VR environment with landscapes to explore perceived ‘vastness’ and found that terminology describing awe actually lead to unreliable survey data; this can occur when a definition of ‘vastness’ is interpreted by the literal visual perception that the area is big and open, opposed to an individual’s self-concept of feeling in the presence of something greater than themselves. Rauhoeft et al. (2015) study demonstrates easily understood definitions and terminology for awe and self-transcendence is important, since there are many ways the experience can be subjectively and empirically described.

Research on awe with virtual environments has combined self-report data with psychophysiological measures to capture the full awe experience. For example, Gallagher et al. (2015) collected self-report, physiological, and neurophysiological measures with the finding that differences in EEG brainwaves exist between the groups of awe-experiencers, and non-awe experiencers. With the human autonomic nervous system responsible for many of our critical body processes like the “fight or flight” response, it is interesting to learn that it also reacts to awe experiences in a unique, powerful way. With awe, we see this through sympathetic nervous system withdrawal and/or increased parasympathetic activation (Shiota et al., 2007, 2011), a phenomenon involving the vagus nerve often regarded as the “tend-and-befriend” response. Chirico et al. (2017) used Skin Conductance Response (SCR), Blood Volume Pulse (BVP), and surface electromyography (sEMG) and found that parasympathetic activation occurred with awe induced using immersive videos compared to 2D videos. It could be that the parasympathetic activation and “tend-and-befriend” response is connected to the reported wellness benefits recorded from awe experiences, with more psychophysiological studies needed to explore this deeper.

### Learning From VR Induced Perspective Shifts

While empirical studies exploring virtually induced awe are emerging, they are still few today. Meanwhile, we can look to studies that explore transformative potential of VR not exclusively related to awe, such as elicitation of perspective shifts. Since awe is thought to generate a shift in self-concept in how a person sees themselves in the world, exploring shifts in self-concept generated through a virtual environment may provide helpful frameworks. Work by Ahn et al. (2014) demonstrates that VR environments, principally the feeling of ‘embodiment’, can result in short and long term behavioral changes through a shift in perspective. Similarly, embodying a superhero in VR increases prosocial behavior, presumably through a shift in self-concept (Rosenberg et al., 2013). In addition to self-report measures, these two studies also added behavioral measures that provided further evidence toward support of a perspective shift. Since induction of such positive emotions and improvement to attitudes are possible through VR (Riva et al., 2016), the implications for changes to an individual’s well-being are encouraging. Several VR environments have been created in the past several decades that may be categorized as a form of “positive technology” with wellness outcomes, and researchers together with designers are exploring this maturing area (Riva et al., 2012; Riva et al., 2016; Baños et al., 2017; Gaggioli et al., 2017; Kitson et al., 2018).

Regarding multimodal sensory devices, VR allows for more than the passive display of audio and visual stimuli. VR becomes active through interaction, which is important since natural body movement in an environment has been positively associated with reported presence levels (Slater et al., 1998). Subjective presence is relevant because the feeling of ‘being there’ (place illusion), and maintaining the illusion that events occurring in the virtual environment are real (plausibility illusion) can lead to realistic behaviors in participants (Slater, 2009, 2011). By enabling the participant to manipulate their environment with body position and interfaces, they may self-select where to navigate. We propose that these actions in the virtual environment feel as though they may be real, leading to authentic experiences and emotions. Furthermore, the use of interactivity may enable more self-relevance and generate awe experiences that have a higher degree of presence than immersive video alone. Thus, interactivity and embodiment could play an important role if we were to use behavioral measures with virtually induced awe.

### Evaluation and Validation of Awe Experiences

Awe experiences and their effects should be carefully evaluated. In lab environments, awe is challenging to elicit, because data is often collected with retrospective self-report methods requiring the participant to constantly self-monitor. If biosensors and monitoring equipment inherent in lab setups are also used, these wearable sensors could be distracting along with the need to self-monitor, potentially lowering the intensities of awe (Benedek and Kaernbach, 2011; Silvia, 2012; Silvia et al., 2015). Questionnaires are also subjective and therefore vulnerable to biases and a participant’s desire to be compliant may affect reliability (Paulhus and Vazire, 2007). Participants may have difficulty finding words to describe a complex event of awe, making thematic interpretation of their experiences challenging; a phenomenological approach may be more favorable (Pearsall, 2008). Likewise, knowing that awe occurred as an overall emotional state doesn’t tell researchers much about the specific elicitors that led to awe, i.e., was there a moment or rush of awe, inspired by seeing a specific aesthetically beautiful scene, or from hearing a crescendo of music/inspiring dialog? These moment-to-moment indications of awe and emotion are important in understanding how to design an awe-inspiring environment.

Empirically, there is evidence that awe can be elicited in the lab through a variety of stimuli. Music and video are highly effective and are often accompanied by chills/goose bumps (Panksepp, 1995; Nusbaum and Silvia, 2011; Maruskin et al., 2012; Wassiliwizky et al., 2015; Colver and El-Alayli, 2016; Schoeller and Perlovsky, 2016; Schubert et al., 2016). Video games elicit these emotions (Lazzaro, 2009; Perron and Schröter, 2016), for example, the video game “Journey” (2012) uses perceptual vastness and a sense of awe to encourage players to connect and have emotional exchanges (Ohannessian, 2012; Isbister, 2016).

As mentioned, self-monitoring can interfere with the ability of the participant to be immersed in the experience, thus reducing the intensity of emotion (Potter and Bolls, 2012). This means that self-reports (through surveys) alone may not sufficiently capture the emotional experience. On the other hand, continuous physiological instruments do not require the participant to self-monitor and may complement introspective self-report data. To validate the presence of specific emotions, researchers have been developing psychophysiological sensors and affective correlates specific to the individual participant (Picard, 2010). If we understand the moment-to-moment attributes of awe, psychophysiological data collection methods of body events via instruments can provide objective, real-time insights. Psychophysiological data complements and enhances the use of traditional self-report psychology methods, particularly when evaluating responses to media forms such as passive video, audio, and interactive games. As non-intrusive data, this helps with ecological validity (Potter and Bolls, 2012), which is important if researchers aim to create virtual experiences that have positive benefits similar to real-life awe-inspiring experiences. There are measures and instruments specific to awe; Grewe et al. (2009) provide solid evidence that the sensation of “chills”/“shivers” are connected to the physiological response of goose bumps as emotional ‘peak’ experiences of awe. In measuring ‘peak’ experiences, Grewe et al. (2009) collected both physiological and real time self-report data to explore physiological correlates of emotion. Novel instruments have been designed to capture fleeting “peak” experiences, such as a video camera for recording goose bumps used by Benedek et al. (2010). Benedek and Kaernbach (2011) found goose bumps are correlated with high emotional arousal, specifically the “being moved” characteristic of awe. Further studies using music and video stimulus with this camera instrument also found goose bumps correlated with “being moved” and a “peak” experience (Sumpf et al., 2015; Wassiliwizky et al., 2017). Schurtz et al. (2011), and Silvia et al. (2015), found goose bumps were correlated with awe; Keltner and Haidt (2003), Campos et al. (2013), and Maruskin et al. (2012) also describe goose bumps with awe-inspiring stimuli. In what may be an ultimate awe-inspiring experience, astronauts have reported goose bumps and chills from seeing the Earth from Space in the Overview Effect. NASA flight engineer and mission specialist Kjell Lindgren describes this:

“I saw this really bright white light coming through the small windows of the Soyuz capsule. I took a peek and saw the beautiful blue and whites of the Earth below, and the curvature of the horizon. Getting to experience the whole disk of the Earth from that point of view, truly for me, it was this breathtaking experience. I got goosebumps.” – in an interview with The Week (Hullinger, 2016)

**FIGURE 1:**
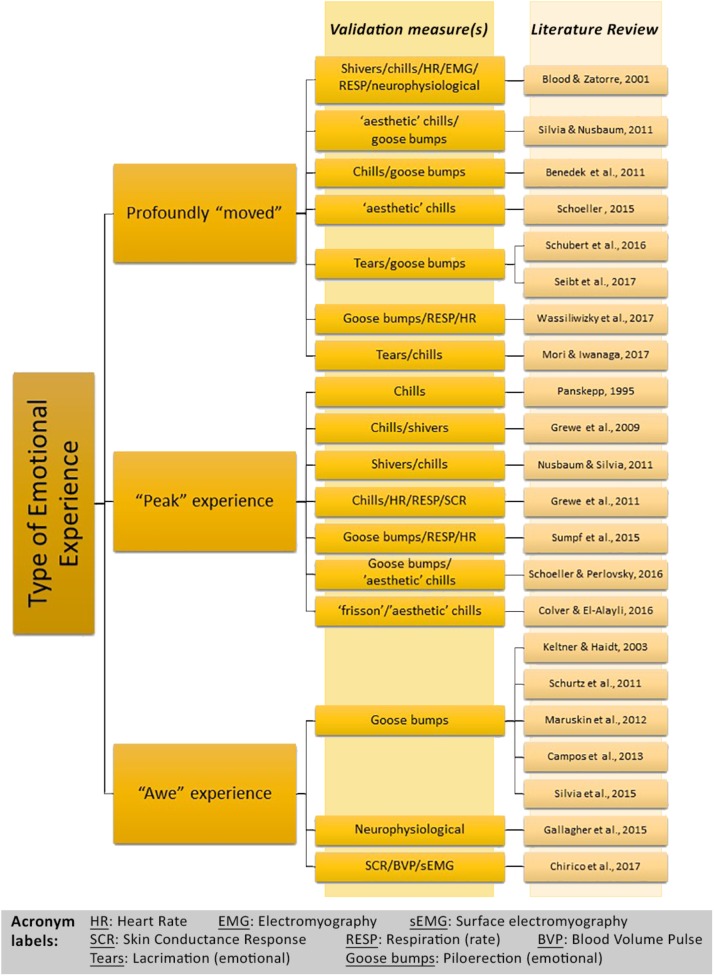
The ways in which awe has been phrased by participants in literature, with the corresponding physiological subjective, and objective validation measures.

The connection of chills and goose bumps to moving, awe-inspiring experiences has been observed by artists and designers over the decades, with distinctions noted between (a) regular chills and goose bump episodes: the feeling of shivers and presence of goose bumps due to cold and thermoregulation; and ‘aesthetic chills’: (b) pleasurable non-thermoregulatory shivering and presence of goose bumps (Schoeller, 2015; Schoeller and Perlovsky, 2016). Aesthetic chills are also cited as ‘frisson’, with these terms interchangeable (Sloboda, 1991; Colver and El-Alayli, 2016). It is important to note the differing terms for the same phenomenon, because awe can be very subjective and described in multiple ways, with more than one concurring emotion present. For example, similar to a “peak” experience, being profoundly “moved” is also correlated with shivers/chills (Blood and Zatorre, 2001; Mori and Iwanaga, 2017) and goose bumps (Seibt et al., 2017). To illustrate this, Figure 1 presents how the experience of awe, the feeling of being profoundly “moved,” and a “peak” experience commonly overlap with one another and physiological correlations; as shown in the literature, there have been multiple ways of describing this phenomenon that potentially share epistemic traits. In an effort to accurately validate emotions, physiological instruments like a goose bump recording instrument and other sensors can be used with the artificial intelligence to differentiate awe and other concurring emotions (Quesnel et al., 2017). As reported in literature, some individuals haven’t actually experienced goose bumps in response to an emotional or aesthetic stimulus (Goldstein, 1980; Sachs et al., 2016; Neidlinger et al., 2017). Additionally, there is mixed evidence regarding the potential role of gender for episodes of goose bumps/chills/shivers: While most studies involving goose bumps/chills/shivers do not report gender as a factor, two previous studies have shown that both male and females have equal episodes of goose bumps/chills/shivers (Goldstein, 1980; Grewe et al., 2009), whereas two other studies observed gender effects, in that females showed more episodes of goose bumps/chills/shivers than males (Panksepp, 1995; Benedek and Kaernbach, 2011). However, a limitation of these studies is that females might be more likely to report chills in a group/social setting as per Panksepp (1995) study, and there was a significant disproportion of 43 female to 7 male participants in Benedek and Kaernbach (2011) study. To address these issues in the current study, we used individual post-experimental interviewing to avoid potential group effects and aimed for a gender-balanced participant sample.

For our study, we placed importance on collecting physiological data in the form of goose bumps that may illustrate the moment-to-moment experience of awe and collect self-report questionnaire data and interview data to provide further insight into the phenomenon.

## Research Questions

This study was designed to provide insight into three research questions (RQs):

**(RQ1) To what degree can interactive VR generate subjective and physiological goose bump experiences of awe?** and;
**(RQ2) What effect(s) do the traits of aesthetic beauty/scale, familiarity, or personalization of the environment have on awe experiences?**

While the findings of (**RQ1)** are quantitative and measured through physiological goose bump readings and awe ratings in self-report questionnaires, the findings for (**RQ2)** are more qualitative in nature, requiring analysis of interview data. (**RQ1**) informs us about whether our selected emotional validation measures are specific to VR induced awe, and (**RQ2**) informs us how the VR might be awe-inspiring and comparable to real-life awe experiences. It should be noted that it is not an objective of the study to compare different virtual environments for awe elicitation, but rather investigate individual traits of the environments. Through qualitative exploration, we analyze the individual participant statements to explore whether specific features such as vastness, beauty, scale, and familiarity are correlated with self-reports of awe and/or physiological goose bumps.

We also explore possible relations between personality traits and potential experiences of awe, as there is a question of whether personality factors are predictors for awe experiences. “Openness,” one of the domains used to describe personality in the Five-Factor Model, has been found to be connected to profound emotional responses over the other personality factors of Agreeableness, Conscientiousness, Extroversion, and Neuroticism (Silvia et al., 2015). Studies have shown “Openness” to be correlated with lab-induced awe (Bride, 2016), and Openness correlating with “frisson”/”chills” from music (Nusbaum and Silvia, 2011; Colver and El-Alayli, 2016). Cultural differences, physical location, daily stressors, willingness to participate, and education level may influence personality traits, which in turn may impact ability to experience awe. As a result, correlations between personality traits and awe may not have good generalizability. In our study, we explore whether personality factors predict awe experiences.

While the measure of ‘absorption’ is often collected in studies (Silvia and Nusbaum, 2011; Elk et al., 2016), we opted to use a comparable measure specific to passive and interactive media: Immersive Tendencies Questionnaire (ITQ: Witmer and Singer, 1998). Items on the ITQ are similar to those on the Tellegen Absorption Scale (TAS: Tellegen and Atkinson, 1974). The TAS and ITQ both utilize data on how likely participants are to be absorbed or immersed, to lose track of time, and to be so involved they are unaware of things happening around them (Parsons et al., 2015). We explore correlations between higher scores in Immersive Tendencies and increased awe ratings or goose bumps.

Previous studies found that experiences in nature and with art (museums, music) can be awe-inspiring (Keltner and Haidt, 2003; Shiota et al., 2007); these studies identified aesthetic beauty/scale as awe elicitors, yet there is a question of how familiarity and personal relevance are factors of an awe experience. Therefore, we propose a qualitative exploration of aesthetic beauty/scale, familiarity, and ability to personalize an environment as potential awe elicitors. It is unknown how many awe elicitors are in a VR experience, so we explore introspective interviews for any arising themes. We look to this introspective data for themes concerning the usability of VR, as the technology itself and its navigation interface may affect awe.

To explore if females would report more goose bumps/chills/shivers as reported in some studies (Panksepp, 1995; Benedek and Kaernbach, 2011) but not others (Goldstein, 1980; Grewe et al., 2009), we included a research question **(RQ3): what effect does gender have on ratings of awe, and rates of goose bumps?** We predicted that if there was an effect, females should show higher incidences of goose bumps/chills/shivers.

## Materials and Methods

### Participants

Sixteen participants, 10 males and 6 females, a mean of 27.3 years of age, were recruited from undergraduate programs at a Canadian University and through a local VR meetup. All participants voluntarily took part in the experiment, and student participants received course credit. None were monetarily reimbursed. This study was carried out in accordance with the recommendations of the 2nd edition of the Tri-Council Policy Statement: Ethical Conduct for Research Involving Humans (TCPS 2), through Simon Fraser University Office of Research Ethics (ORE). The protocol was approved by the Simon Fraser University ORE prior to data collection (REB #2012c0022), with all consent forms, procedures, and methods compliant to the 2nd edition of the TCPS 2. All participants gave written informed consent in accordance with the Declaration of Helsinki and received identical instructions.

### Stimuli and Apparatus

**FIGURE 2:**
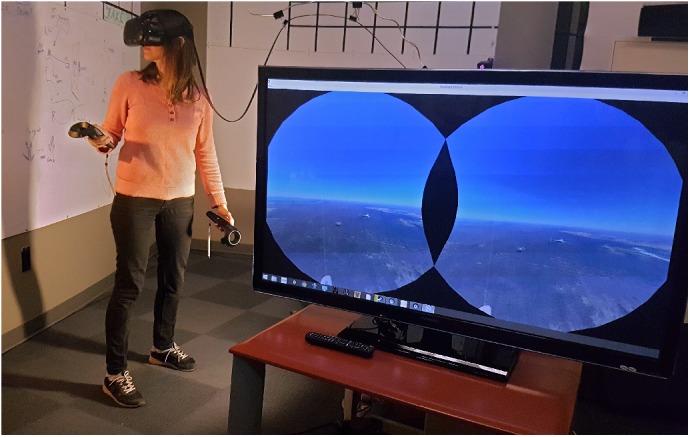
A participant from the pilot experiment (who does not contribute to this studies’ dataset) is wearing the VR headset and navigates Earth with handheld controllers. Informed and written consent was obtained from the pilot participant depicted in the photo.

Provided that the most common awe elicitors are found in nature scenes and as visuals of the Earth from space (Keltner and Haidt, 2003; Shiota et al., 2007; Grewe et al., 2011; Gallagher et al., 2015; Silvia et al., 2015; Yaden et al., 2016; Gordon et al., 2017), we opted to use a VR environment that allows for the appraisal of the Earth’s landscapes, cityscapes, and a view of the planet from Earth’s orbit. Each of these environments incorporates some of these awe elicitors, which were determined by participants in our pilot study, and while we do not aim to compare environments as more or less awe-inspiring from one another, we do intend to provide a qualitative analysis exploring moments within the environments that may indicate awe. To evaluate the full capacity of VR in eliciting awe, our stimulus also included interaction with the environment. For the most accurate and high-resolution representation of the Earth that also involves interactive modes, the application “Google Earth VR” was selected as the stimulus. The application features thousands of locations in stereoscopic 3D, with imagery displayed in real-time (Käser et al., 2016; Käser et al., 2017). The stimulus is interactive through tracked head position and two handheld controllers, allowing the user to fly through the environment (Figure 2). As an existing system, Google Earth VR was selected for this study because we required a complete, “whole” Earth model of excellent resolution that enabled participants to recognize landmarks, and to get close to destinations of the user’s choice. At the time of this study, no other Earth model in VR reached the level of realism and resolution that Google Earth VR could. The navigation via hand controller as input device worked by using a trigger button (right hand) to point, select, and drag the environment; a touch pad enabled forward and backward movement (right hand); and a touchpad (left hand) enabled a vertical or horizontal orientation on the Earth. At the time of execution, Google Earth VR had only been publicly available for 7 days, and none of the participants had tried it.

#### Virtual Environment Locations and Order

There are four environments: (i) Non-interactive Color Tour; (ii) interactive Vancouver, Canada; (iii) interactive Mount Everest, in the Himalayas; (iv) interactive place of the participant’s choosing (self-selection). Participants experienced all four environments for 5 min each, in the same fixed order:

##### (i) Color tour

**FIGURE 3:**
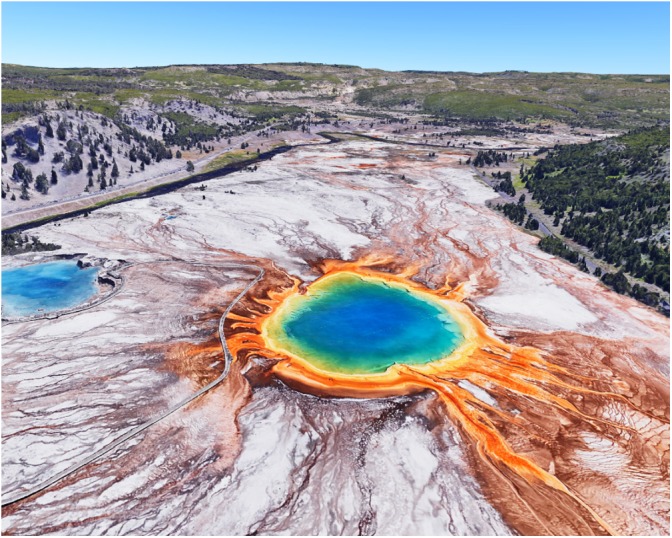
Color Tour environment, as seen on the external monitor of the VR display.

A 5-min tour by Google Earth VR’s development team acclimatizes the immersant to VR, and doesn’t require hand controllers- an immersant may look around but they cannot navigate; no navigation skills needed. Potential awe-elicitors: Locations may have aesthetic beauty and scale effects, like colorful natural landscapes, and vibrant buildings; some may be familiar to participants (Figure 3).

##### (ii) Vancouver, Canada

**FIGURE 4:**
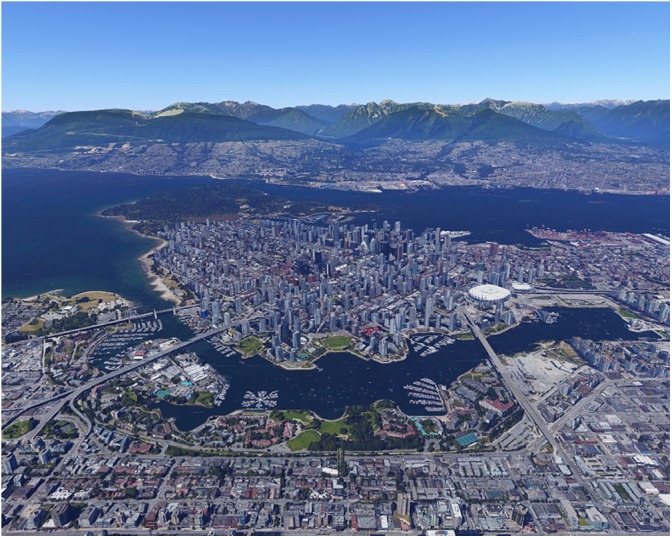
Vancouver, Canada environment, as seen on the external monitor of the VR display.

Potential awe-elicitors: In addition to its aesthetic natural beauty, Vancouver was selected to provide familiarity, since this experiment was physically situated in the Vancouver area. The familiarity enabled participants to be able to navigate easily (Figure 4).

##### (iii) Mount Everest, in the Himalayas

**FIGURE 5:**
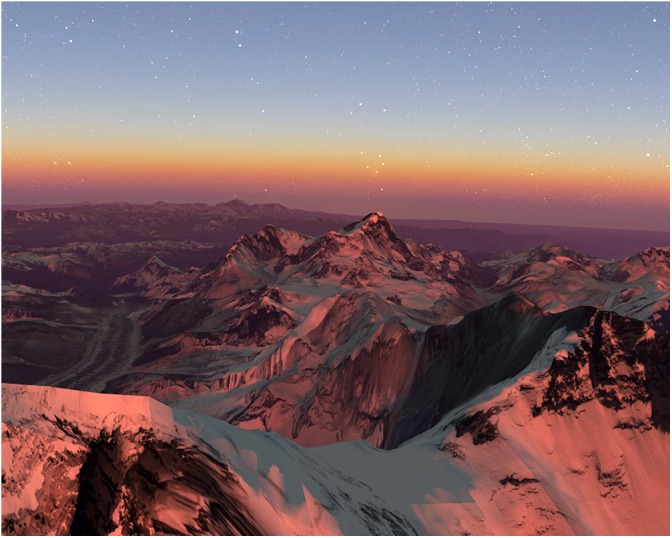
Mount Everest environment, in the Himalayas, as seen on the external monitor of the VR display.

Potential awe-elicitors: Aesthetic supernatural vista and beauty and scale/vastness effects may be present with the participant positioned at summit. This vantage point was not familiar to participants in the pilot study and required more navigation skills (Figure 5).

##### (iv) A place of the participant’s choosing (self-selection)

**FIGURE 6:**
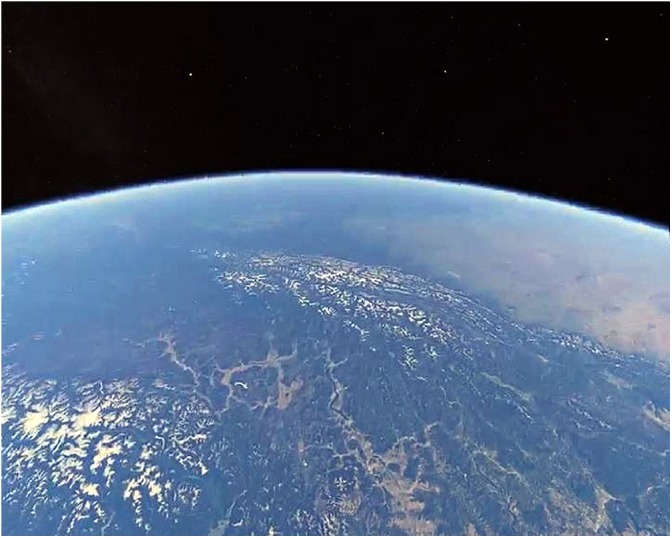
A self-selected environment where the participant orbits the Earth from Space, as seen in the external monitor of the VR display.

Any location of the participant’s choosing; Teleportation navigation was activated, enabling travel along the ground or orbiting the Earth, requiring more navigation skills than previous environments. Participants were free to do whatever they liked during this final personalized aspect of the study. Potential awe-elicitors: aesthetic beauty, scale/vastness, familiarity/personalization, or emerging traits (Figure 6).

All environments included views of the Earth from Space, if the participant chose to orbit during navigation.

We opted to have the participants undertake the four virtual environments in a fixed order due to observations in our pilot study with 11 participants. We initially introduced the environments in a counterbalanced order, however, participants that didn’t experience the controller-free Color Tour first struggled considerably with getting used to simultaneously wearing the VR equipment, being in a VR environment, and coordinating the hand controllers. Three of these participants mentioned mild motion sickness in the first minutes of being in VR, explaining that it happened when they “lost control” of their locomotion ability. Pilot participants who undertook the Color Tour before the other environments appeared to ‘ease’ into the more interactive environments with fewer reported mistakes with the interfaces. The other issue with a counterbalanced order of four environments is that the qualitative data collection in this study was extensive, and time constraints limited the number of participants that could be used. Instead of utilizing a large sample size, we opted to use a smaller sample size and focus in depth on the details of these participants’ experiences. We chose to present the Vancouver scene second as it was more familiar and thus harder to get lost in than the subsequent Everest scene. Based on pilot testing, we expected the self-selected scene to require the most navigation skills, and consequently presented it last to reduce usability issues.

#### Settings and Equipment

Each environment lasted 5 min. Participants could explore as they chose in all but the Color Tour. The choice to remain where they were and not explore was open to them. The stimuli contained 3D audio and instrumental musical score. An audio track through noise-canceling headphones was intended to block out outside sounds from the lab setting and enhance sensory immersion, and to provide continuous music. We disabled the “comfort mode,” a feature designed for reduction of motion sickness through blurring of the peripheral vision while moving. The overall scale of the environment was set to “human scale.”

The stimulus was presented stereoscopically on a 2016 model HTC Vive VR system consisting of a head-mounted display (2160 × 1200 total resolution, 1080 × 1200 per eye, 90 Hz refresh rate at 110-degree diagonal field of view), with cables to the headset draped overhead to avoid drag on the participant’s head. Audio was presented on Sennheiser noise-canceling headphones. The computer contained an Intel i5 6600 CPU and a GeForce GTX 970 graphics card.

### Measures

#### Goose Bump Recorder Instrument

**FIGURE 7:**
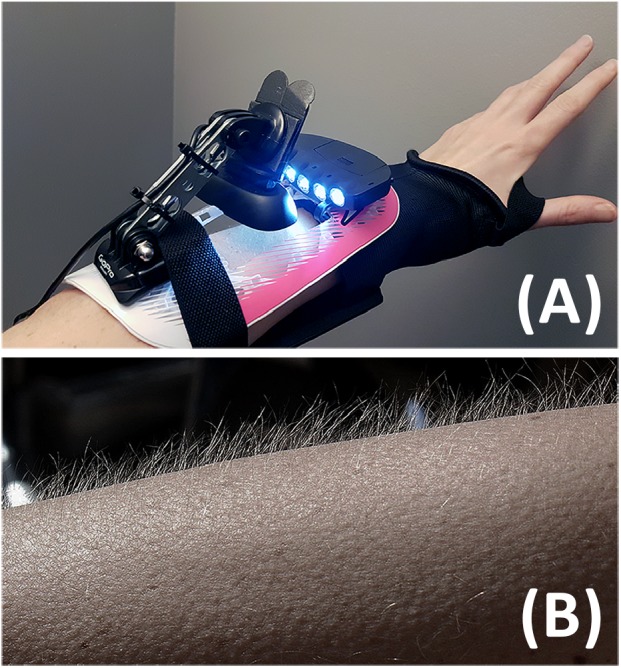
Image **(A)** depicts a model wearing the Goose Bump Recorder Instrument on the arm; **(B)** depicts a typical presentation of the models’ goose bumps.

In exploring (**RQ1**), we determine the frequency of goose bumps for each participant. To record these, we designed an instrument adapted from Benedek et al.’s (2010) ‘goosecam’ consisting of a Logitech HD C270 webcam. The focus ring was modified to allow macro recording of a 5 cm × 5 cm patch of forearm skin, with 3 LEDs at a 15-degree angle to cast unidirectional light onto the skin (Figure 7). This angle captured changes to skin texture. The camera was fixed onto curved fiberglass with soft foam on the skin side. Two elastic straps secured the instrument in place on the non-dominant arm. The instrument was designed to accommodate VR hand controllers. Video from the camera was analyzed via high-contrast filters to determine the presence of goose bumps on the skin. Video was synced to a screen recording of the participant’s VR headset output (via Open Broadcaster Software). To view the videos side by side, the videos were imported into Adobe Premiere and observed on a timeline. Through syncing the instrument recording to the VR screen capture as time series, we see the “moment-to-moment” temporal correlations in the participant’s VR view, and any goose bump episodes that may occur. This allows researchers to directly see and evaluate both the stimulus and participant’s response.

#### Questionnaires

##### Experience questionnaire

To address (**RQ1**), we designed a post-treatment questionnaire (“Experience Questionnaire”) to collect participants’ awe ratings of the VR experience. Participants were asked to rate their experience on a visual analog scale of 0–100 across all four environments (the overall VR experience) with respect to awe, wonder, curiosity, and humility, with definitions for each provided by Gallagher et al. (2015), as seen below:

###### Awe

A direct and initial feeling when faced with something incomprehensible or sublime. Specification: Captured by view/ drawn to phenomenon; elation; experience-hungry, overwhelmed, scale effects, sublime, surprise.

###### Wonder

A reflective feeling one has when unable to put things back into a familiar conceptual framework. Specification: Inspired; Perspectival shift; Nostalgia; Unity with whole; Unity of external; Responsibility.

###### Curiosity

Wanting to know, see, experience, and/or understand more. Specification: Interest/inquisitiveness; Experience-hungry; Intellectual appreciation.

###### Humility

A sense one has about one’s relation to one’s surroundings or of one’s significance. Specification: Responsibility; Unity with whole; scale effects.

Participants were presented with the definition and question about their experience on this questionnaire:

“Please note the following definition- When we use the word AWE, we mean: a direct and initial feeling when faced with something incomprehensible or sublime.” “Did you feel AWED by the experience?”

A visual analog scale was presented from “0” (Not at all awed) to “100” (Definitely awed).

To explore the potential role of personality and immersive tendencies on the experience of awe in VR, we use additional questionnaires:

##### Personality traits questionnaire

The 44-question Big Five Inventory of personality, or BFI (John et al., 2008) consists of questions with a five-point Likert scale, with the minimum score possible being 44 points, and maximum possible being 220 points. Questions were broken into 5 traits: Agreeableness (9 questions for a maximum total 45 points), Conscientiousness (9 questions for a maximum total 45 points), Extroversion (8 questions for a maximum total 40 points), Neuroticism (8 questions for a maximum total 40 points) and Openness (9 questions for a maximum total 45 points).

##### Immersive tendencies questionnaire (ITQ)

The immersive tendencies questionnaire (ITQ) is an 18 question survey, presented on a 7-point Likert scale (Witmer and Singer, 1998). It consists of the subscales: “Involvement,” the tendency to become involved in activities; “Focus,” the tendency to maintain focus on current activities; and “Games,” the tendency to play video games.

##### Demographics questionnaire

To learn more about our sample population we used the following general demographics questions:

1. how many times the participant experienced VR before the experiment (as a number);
2. the participant’s experience with 3D games as a visual analog scale from 0 (no experience) – 10 (expert);
3. age in years, and;
4. gender.

#### Introspective Open-Ended Interviews

To explore (**RQ2**), we used qualitative methods of open-ended interviews and observations of the participants during the experiment. With an experience of awe being complex, and awe elicitors in a VR environment largely unknown, we opted to use the most detailed descriptions of awe-inspiring experiences in the literature. These descriptions were the analysis of astronauts’ awe experiences placed into 34 consensus categories by Gallagher et al. (2015). Participant interviews were transcribed and statements from the interviews were matched within four definitions of awe, wonder, curiosity, and humility, and are found within the 34 consensus categories. To explore how the environment might affect awe experiences and include specific awe elicitors, we looked for repeating categories and statements that occurred during the virtual environments. We recorded themes around the usability of the VR system and navigation interface to better understand the role of interactivity and usability on experiences of awe. Finally, we specifically looked for participants’ referring to their personal history: these statements may provide insight to their ability to become awed or not.

A video camera with audio recording capability was used to capture the participant’s experimental session and interview.

### Procedure

Participants signed informed consent, then were briefed that they would experience VR. They were shown the goose bump recording instrument and told that they may ask questions. The facilitator took care to avoid stating expectations around the VR stimuli. Participants completed the 44-question personality traits evaluation (BFI: John et al., 2008). Next, participants were told that they would enter Google Earth VR, which began with a 5-min tour with VR hand controllers not needed. They were shown and taught the VR headset and controllers before beginning the 5-min tour. This allowed them to seamlessly enter the next environment without the facilitator disrupting their experience.

**FIGURE 8:**
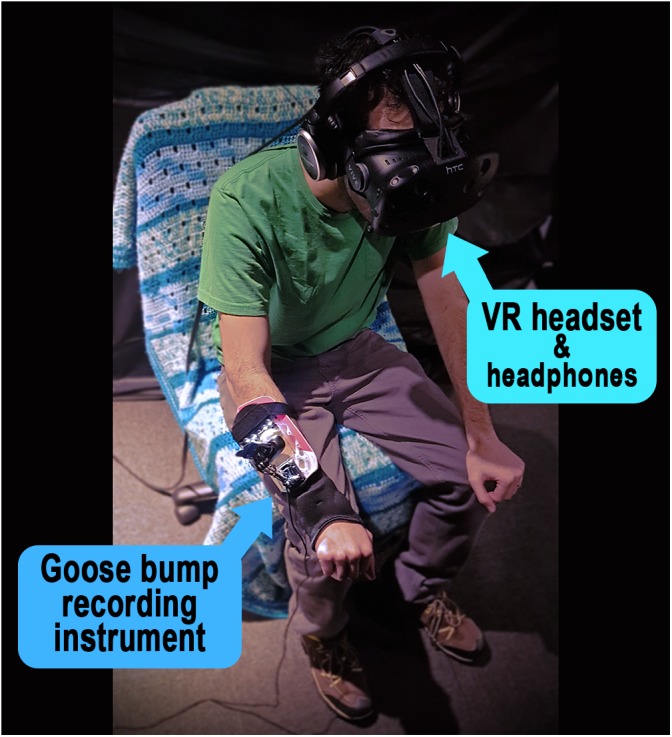
The VR equipment setup with headset, noise-canceling headphones, and goose bump recording instrument. Informed and written consent was obtained from the participant from the pilot experiment depicted in the photo (who does not contribute to this studies’ dataset).

The goose bump recording instrument was placed on their non-dominant arm. Participants were fitted with the VR headset and headphones, with adjustments made by the facilitator for comfort (Figure 8). The presentation of the stimuli then began with the Color Tour. Once the tour was finished, participants were handed the controllers and began their exploration in Vancouver. Upon completion of 5 min in exploring this environment, participants went to Mount Everest for 5 min. After this third environment, participants were told to navigate to a destination of their choosing, anywhere in the world, for a final 5 min.

Once their self-selected environment was complete, VR equipment was removed, and participants were given the Experience Questionnaire, followed by the demographics questionnaire, and final questionnaire on their Immersive Tendencies (ITQ: Witmer and Singer, 1998). Upon survey completion, an open-ended interview was conducted. Questions directed at participants included an indirect inquiry about the participant’s sense of time (“You’ve spent 20 min in VR today, how do you feel?”), a question directed at their experience (“was there anything you’d like to talk about from that experience?”), a question about anything that stood out (“did anything stand out to you?”), and a question directed at their self-selection (“What is the significance of the place you chose to travel to?”). No other specific questions were asked, instead the interview was open-ended so the participant could talk about their feelings in the moment. The facilitator added these responses to observations made during the participant’s session, such as whether they turned around and utilized the full 360 degrees of the headset, how they handled the controllers (with ease, or not at ease), how vocal the participant was, and how enthusiastic/animated the participant was before, during and after the experiment. The entire experiment lasted 60 min.

## Results

All effects are reported at a 0.05 level of significance. Parametric statistics were used for all quantitative analysis. Unless specified, assumption of normality is confirmed.

### Subjective Awe Levels

**FIGURE 9:**
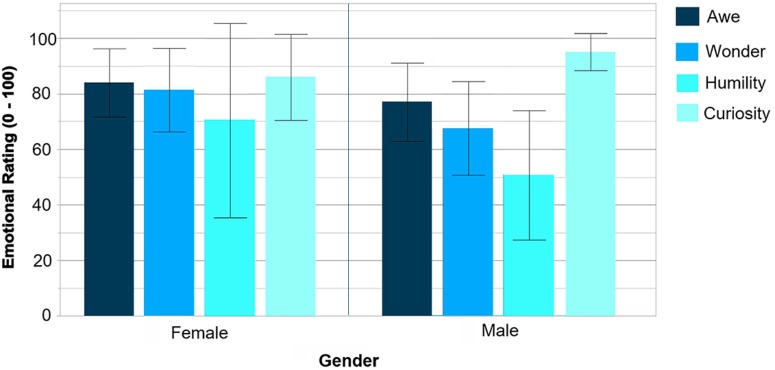
Emotional ratings across all four environments by gender. Each error bar is constructed using a 95% confidence interval of the mean. For females: awed (*M* = 84.0, *SD* = 11.7), wonder (*M* = 81.3, *SD* = 14.3), humility (*M* = 70.5, *SD* = 33.3), and curiosity (*M* = 86.0, *SD* = 14.8). For males: awed (*M* = 77.1, *SD* = 19.7), wonder (*M* = 67.6, *SD* = 23.5), humility (*M* = 50.7, *SD* = 32.5), and curiosity (*M* = 95.1, *SD* = 9.3).

Overall across the four environments, participants rated their emotional engagement for feeling awe, wonder and curiosity fairly high (averaging above 70 on a 0–100 scale), as summarized in Figure 9. To support this finding, we use a threshold of 10–100 for determining heightened emotions, as per Gallagher et al. (2015, p. 91).

Overall ratings of awe were high across all individuals (*M* = 79.7, *SD* = 17.1). Although females (*M* = 84.0, *SD* = 11.7) reported slightly higher awe ratings than males (*M* = 77.1, *SD* = 19.7), this trend did not reach significance, *t*(14) = -0.78, *p* = 0.449, *d* = 0.43.

Similarly, females showed a non-significant trend toward higher ratings of wonder (*M* = 81.3, *SD* = 14.3) than males (*M* = 67.6, *SD* = 23.5), *t*(14) = -1.28, *p* = 0.221, *d* = 0.70.

Humility ratings were overall a bit lower than the other ratings (*M* = 58.1, *SD* = 33.2), with males showing a non-significant trend toward lower humility ratings (*M* = 50.7, *SD* = 32.5) than females (*M* = 70.5, *SD* = 33.3), *t*(14) = -1.17, *p* = 0.261, *d* = 0.60.

Finally, curiosity ratings were overall high (*M* = 91.7, *SD* = 12.1), with males showing slightly higher curiosity ratings (*M* = 95.1, *SD* = 9.3) than females (*M* = 86.0, *SD* = 14.8). Again, this trend did not reach significance, *t*(14) = 1.52, *p* = 0.152, *d* = 0.74.

**FIGURE 10:**
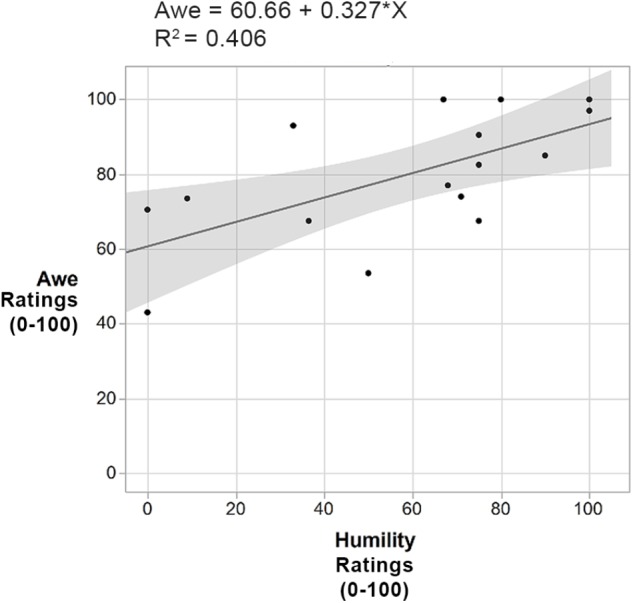
Awe and humility ratings correlational diagram.

Using bivariate correlation analysis, results indicated that participants whose rankings of humility were high, also had closely correlated high rankings of awe (*r*^2^ = 0.41, *F*(1,14) = 9.6, *p* = 0.008), see Figure 10. None of the other ratings were correlated. The positive correlation between awe and humility is of interest when we explore the qualitative findings, specifically the concept of ‘*small-self*’ / diminished perceived size as a self-transcendent quality (Bai et al., 2017).

**To summarize the subjective awe findings**, we did find support for our (**RQ1**) in that the four different interactive VR environments yielded an average awe rating of 79.7 out of a 0–100 scale, indicating that interactive VR can indeed induce awe.

### Goose Bumps Occurred in Almost Half of the Participants; Most Occurred in the Final Self-Selection VR Environment

43.8%, or 7 of the 16 participants experienced goose bumps as detected by our goose bump recording instrument throughout the four environments, which is consistent with previous research (Benedek and Kaernbach, 2011; Sumpf et al., 2015; Wassiliwizky et al., 2017). With equal variances confirmed and using a *t*-test, there was a significant trend for females experiencing more goose bump occurrences (*M* = 1.3, *SD* = 1.4) than males (*M* = 0.3, *SD* = 0.5) *t*(14) = -2.21, *p* = 0.04, *d* = 1.0. This finding demonstrates that females are slightly more likely to have goose bumps than males (**RQ3**).

Interestingly, 64% of the goose bump occurrences from all participants occurred in the final self-selection VR environment, where participants traveled to a location of their choosing. The qualitative interviews provide more specifics into this finding and are discussed in detail in the Section “Most Goose Bumps Occurred During the Self-Selected Environment.”

### Awe Ratings Were Positively Correlated With the Occurrence of Goose Bumps

**FIGURE 11:**
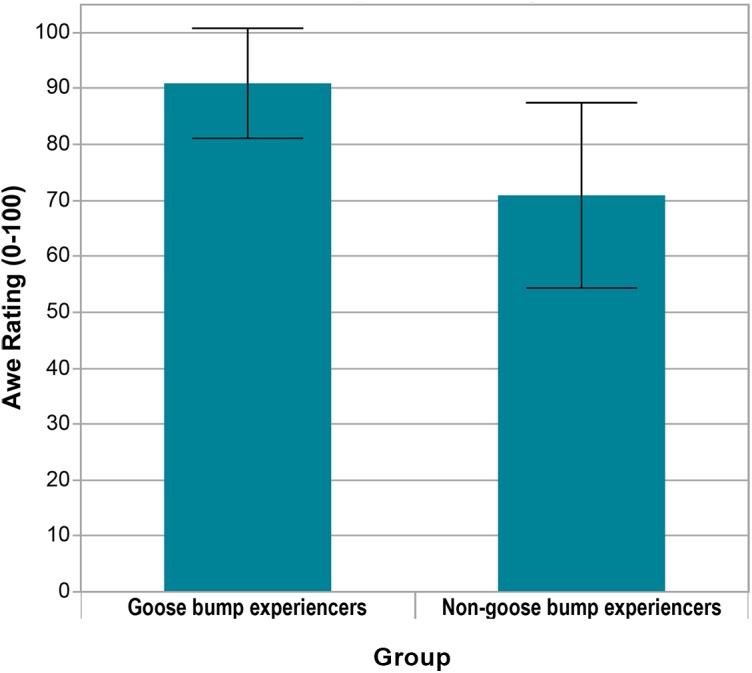
Awe ratings between the groups ‘goose bump experiencers’ (*N* = 7, *M* = 90.9, *SD* = 9.8), and ‘non-goose bump experiencers’ (*N* = 9, *M* = 70.9, *SD* = 16.6).

To explore the relationship between the occurrence of goose bumps and awe ratings, participants (*N* = 7) who had goose bumps were sorted into a “Goose bump experiencers” group, and those who did not have goose bumps (*N* = 9) were sorted into a “Non-Goose bump experiencers” group. When the goose bump-experiencers were separated from non-goose bump experiencers, there a significant effect of the presence of goose bumps on the ratings of awe, see Figure 11. Awe ratings were significantly higher for goose bump experiencers (*M* = 90.9, *SD* = 9.8) than for non-goose bump experiencers (*M* = 70.9, *SD* = 16.6), *t*(14) = 2.82, *p* = 0.01, *d* = 1.42. This indicates a correlation between the occurrence of goose bumps and higher awe ratings and supports the literature that goose bumps are a probable physiological indication of awe (Keltner and Haidt, 2003; Schurtz et al., 2011; Maruskin et al., 2012; Campos et al., 2013; Silvia et al., 2015). This means that use of a goose bump recording instrument may be valuable in collecting data on moment-to-moment indications of awe.

To summarize the findings for (**RQ1**), data indicate that the interactive VR stimuli generated subjective (through awe ratings) and physiological (through goose bumps) indications of awe. This finding suggests there is merit in collecting both subjective ratings and physiological data for the evaluation of awe, as both methods demonstrate valuable insight into the awe phenomenon; ratings represent an ‘overall’ sense of awe, and physiological goose bumps show ‘moment-to-moment’ indications.

### Personality Traits Are Not Clearly Correlated to Goose Bump Occurrences and Subjective Awe Ratings

**FIGURE 12:**
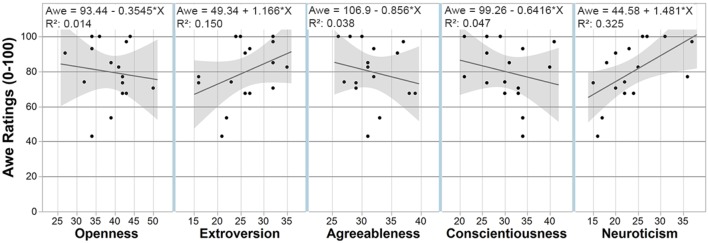
Personality traits vs. awe ratings correlational diagram.

Prior evidence has demonstrated that Openness as a trait may be correlated with a higher incidence of awe (Silvia et al., 2015; Bride, 2016), and awe with goose bumps/chills (Silvia and Nusbaum, 2011; Colver and El-Alayli, 2016). Here, bivariate analysis was used to test if the BFI personality traits significantly predicted participants’ ratings of awe (see Figure 12). However, we found no evidence that Openness as a trait predicts higher **awe ratings** in this study. Openness, Agreeableness, and Conscientiousness showed non-significant negative correlations with awe ratings (all *p*’s > 0.05). Extroversion was found to be slightly but not significantly positively correlated with awe ratings, (*r^2^* = 0.150, *F*(1,14) = 2.48, *p* = 0.138). However, Neuroticism had a slight positive correlation with awe ratings (*r^2^* = 0.325, *F*(1,14) = 6.73, *p* = 0.02), but the medium effect size indicates limited linear relation between the two variables.

No correlation between **goose bump counts** and any of the BFI personality traits were found (all *p*’s > 0.05).

### No Correlations Between Immersive Tendencies and Goose Bump Frequency or Subjective Awe Ratings

Correlation analyses showed that immersive Tendencies scores did not appear to have any effect on **high responses** **of awe** or physiological **goose bump** **incidences** (all *p*’s > 0.05). Thus, participants with higher Immersive Tendencies didn’t appear to be more likely to experience higher awe in VR, at least not in the current study with the small participant sample.

### Introspective Open-Ended Interviews Show Specifics of Experience

To address (**RQ2)**, we used introspective data from the interviews to gain a deeper understanding how the traits of aesthetic beauty/scale, familiarity, or personalization of the environment relates to the participants’ experience. Statements were classified into categories of awe and usability of technology by one trained researcher who also conducted the open-ended interviews, using pre-defined coding units and themes. Awe categories were obtained, and validated from Gallagher et al. (2015, p. 29).

#### Participant Statements Within the Categories of Awe

**FIGURE 13:**
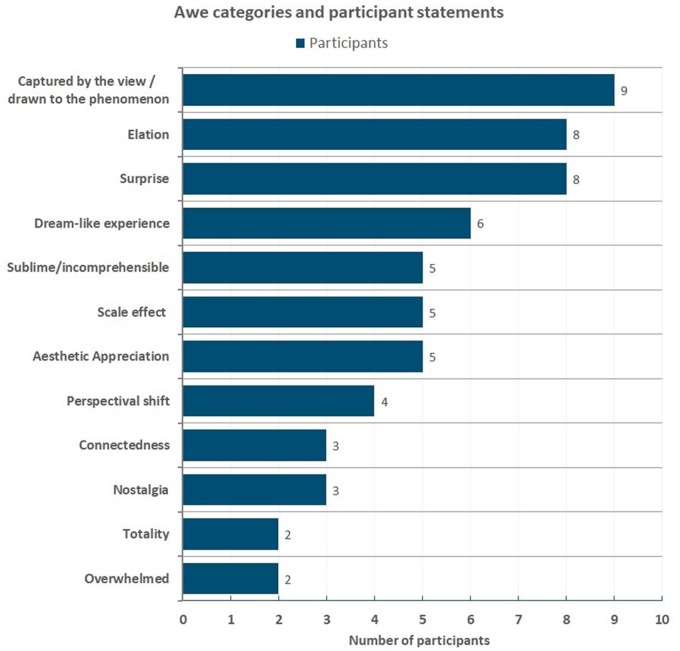
12 categories of astronauts’ awe experiences based on Gallagher et al. (2015) textual analysis. Participant statements are sorted into the awe categories, with the frequency of participants noted who made statements in each category.

All participants had at least two statements during the interviews that could be categorized into one of Gallagher et al. (2015) consensus categories of awe. Given that the categories were generated based on astronauts’ experiences in space, and since we used a VR environment that allowed for viewing Earth from space and Earth at ground level, we anticipated some similarities between statements. However, as the astronauts’ experience of viewing the actual Earth from space is a considerably powerful experience involving specialized training and danger, we expected participants to report less intense experiences than the astronauts. Figure 13 summarizes the categories and frequency of statements by participants.

Although Gallagher et al. (2015) determined 34 categories of awe and wonder based on descriptions by astronauts, we found that our participant statements fit into a subset of just 12 of these 34 categories:

(1) Aesthetic Appreciation
(2) Captured by the view / drawn to the phenomenon
(3) Connectedness (feeling connected with something without losing distinctness)
(4) Dream-like experience (feeling of unreality, abstract feeling)
(5) Elation
(6) Nostalgia
(7) Overwhelmed
(8) Perspectival shift (internal change of [moral] attitude)
(9) Scale effect (feelings of the vastness of the universe or one’s own smallness/ insignificance)
(10) Sublime/incomprehensible
(11) Surprise
(12) Totality (wholeness of what is experienced; big picture)

It is possible that a broader participant sample and a more comprehensive interview technique, such as neurophenomenology or micro-phenomenology would have enabled us to dig deeper into the experiences and associate participant statements into more categories. For the style of open-ended interviews that we used for the current study, however, our participant interview statements did not fit into any of the remaining categories defined by Gallagher et al. (2015):

(13) Change (internal or bodily change)
(14) Contentment (tranquility, feeling relaxed or at peace)
(15) Disorientation
(16) Emotional (general emotional feeling or arousal)
(17) Experience-hungry (wanting more of a particular experience)
(18) Exteroceptive intensive experiences (sensory overload, silence)
(19) Floating (bodily, feelings of weightlessness)
(20) Floating in void (not related to weightlessness)
(21) Fulfillment
(22) Home (feeling of being at home)
(23) Inspired
(24) Intellectual appreciation (for order, analysis, complexity)
(25) Interest/inquisitiveness
(26) Interoceptive intensive experiences
(27) Joy (feeling of happiness)
(28) Perspectival (spatial) change
(29) Peace (conceptual thoughts about)
(30) Pleasure
(31) Poetic expression
(32) Responsibility (toward others)
(33) Unity of external (earth, universe, people on earth, interrelatedness)
(34) Unity with whole (feeling of oneness with; holistic feeling)

We wish to note that category #5 “Elation,” #16 “Emotional (general emotional feeling or arousal),” #27 ‘Joy (feeling of happiness),” and #30 “Pleasure” overlap each other considerably. All four categories describe a particular positive affect, or overall emotional state. While some participant statements did reflect these three categories, we chose to focus on #5 “Elation” which tended to be more specific. Because of the overlap, we took a conservative approach to the analysis of under-categorizing, opposed to over-categorizing statements, thus helping to avoid potential mis-categorization.

As illustrated in Figure 13, the most common statements fit into the category of “Captured by the view/drawn to the phenomenon,” mentioned by 9 of the 16 participants (56%). This isn’t surprising given part of the operational definition of awe contains the specific categories of: Captured by view/drawn to phenomenon; elation; experience-hungry, overwhelmed, scale effects, sublime, and surprise. Some examples of feeling captured by view/drawn to phenomenon were spoken by participants as they were in the VR environment:

“*I feel like superman, flying above Earth and seeing all these cool things! I’ll never get enough of this view! Look!*” P08, female

On the other hand, some participants went to places they would be traveling to in real life and treated the VR environment like a preview. One participant describes being captured by the view:

“*I’m going to London and Japan soon, so I thought I’d enjoy what it will be. London has a lot of landmarks that look really great to see. Japan though, has some natural sights I now want to visit. It draws you right in, to see that. I feel like my eyes were locked on that.*” P15, female

Another participant describes the difference between a photo of a place, and feeling like they are there in VR while being drawn in:

“*If I saw a photo, I wouldn’t care… But this made me feel it was just real enough to sort of be there, and want to go there more. I felt drawn to that place.*” P14, female

Feelings of elation and surprise were also common among the participants interviews (8 of 16, or 50% of participants for both). Two participants described how the experience could be mood altering:

“*I don’t know what I would feel like (after), but I felt totally happy. Like, nothing can disappoint me now today.*” P05, female

“*This was so amazing. I feel like my week is going to be a lot less stressful. Whenever I think of all the things I have to do, I’ll think of this and how fun it was.*” P14, female Some participants described the delight and elation of seeing familiar places in a new way:

“*This is totally outrageous. I can’t believe what I am seeing… Actually I can. I am seeing my planet but from a totally different way. I can go anywhere, I love this*.” P06, male
“*That was so cool. I could do that forever. It makes me really happy to be able to go home, whenever I want. I’m a small town kid and there’s comfort in that, in seeing it this way, you know?*” P13, male

It should also be noted that these four above statements may also be categorized as #16 Emotional (general emotional feeling or arousal), #27 Joy (feeling of happiness), and #30 Pleasure.

There was some overlap between elation and surprise, with many statements indicating both happened in parallel:

“*I liked the beginning of the experience a lot.” … “Because I didn’t know those places, that they existed. Couldn’t wait to see what was next. They are beautiful. I am really surprised.*” P04, male

Several participants (6 of 16, or 38%) made note that the experience felt dream-like, or like drifting through unreality. One participant voices how he lost track of time:

“*Time just stopped. It felt like daydreaming, when hours pass but it feels like just minutes.*” P09, male

Aesthetic appreciation frequently was heard, with many participants stating their environments were “breathtakingly gorgeous,” “really nice to look at,” and with “lots to see, very beautiful.” Likewise, many statements indicating feelings of sublime/incomprehension were mentioned:

“*I can’t believe what I am seeing. Is that real? It feels real.*” P08, female

Feeling overwhelmed wasn’t commonly reported, but in the two instances it was stated was in the context as being positive:

“*…the type of feeling, of being overwhelmed when seeing earth from high up, is really neat.*” P11, female

Five participants mentioned scale effects, describing the visuals as perceptually vast or large, like massive oceans and coastlines. Two participants mentioned feeling both perceptually and conceptually small compared to the environment:

“*I don’t feel very significant right now. I mean that’s ok, I just feel like there’s a big planet full of people and things out there, and I am just one. It means my problems are actually smaller than I think, in reality.*” P08, female
“*I can’t believe this can exist. It feels much greater than me.*” P01, male

These two comments may be indicative of the ‘*small-self’*, or diminished size phenomenon in an awe-inspiring experience (Piff et al., 2015; Campos et al., 2013; Elk et al., 2016; Bai et al., 2017).

Some participants stated a feeling of connectedness, and a yearning to connect (3 of 16, 19% of participants). We can see with the following statements some examples:

“*That’s my home. That’s my old home, where I grew up. I was… I left when I was nine, I haven’t seen it since. That fence, it is still there, same fence. Can I see a person? Maybe she is there?*” (participant speaking aloud to themselves during the experiment, with “she” later revealed in the interview to be a grandmother). “*I think I need to… I haven’t talked to her, my sister, or grandmother in a long time. They are there still. I should talk with them, over video, you know?*” (stated during the interview). P02, male
“*I feel like I could do this for a long time, and just get lost in it. Imagine if you could connect with other people there, like we do in a dream, people you know. I feel like sharing that.*” P16, male

Relating to connectedness was some statements around experiencing nostalgia. Three participants mentioned nostalgia in their interviews, and each participant experienced nostalgia when looking upon a childhood and ancestral home, like the following:

“*It doesn’t look like much changed, from the photos. It’s very nostalgic.*” P10, male

Few participants appeared to have statements that could be categorized as perspectival shifts. Included among the four participants who mentioned potential changes to their moral attitude we see: one possible change to how they perceive the environment; one perception that problems may be small in proportion to others; and two perceptions that individuals in other places have a challenging way of life that should elicit empathy.

Totality was mentioned just twice in the interviews, but both related to the sight of seeing Earth from space:

“*It was amazing to see the whole planet from this perspective, makes feel free, and able to see the whole of everything.*” P14, female

The lack of comments attributed to totality may be because most participants opted not to orbit the Earth, but rather looked for more familiar features closer to ground level.

#### Most Goose Bumps Occurred During the Self-Selected Environment

**FIGURE 14:**
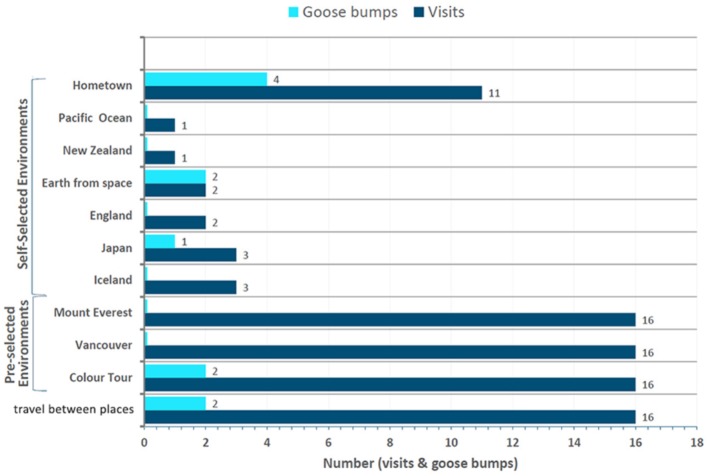
The total number of visits to each place inside the VR environments across all participants, and the total number of goose bump occurrences (if any) for each place. Please note that all participants experienced the pre-selected environments of Color Tour, Vancouver, and Mount Everest; all participants engaged in travel between places by navigating from place to place and around the environment. Some participants elected to travel closer to the ground, while others orbited above Earth. Some participants experienced more than one occurrence of goose bumps during the experiment.

Most of goose bumps occurred during the final self-selection phase of the experiment (64%), with the most common self-selected destinations, as explained by the qualitative data, being participants’ hometowns where family and friends are, or places participants wished to visit (Figure 14). Familiar environments appear to draw an equal amount of awe-inspiring feelings as environments that are unfamiliar. In allowing the participant to personalize their experience, our aim was to enable the participant with agency and ownership of their experience, a capability many participants stated they enjoyed. Personalization of an experience and its correlation to awe is compelling, with many participants stating this was an overwhelmingly positive experience; additional introspective findings as seen in the interview excerpts also reveal themes of social connection during the self-selected environments.

#### Controller-Based Interaction May Impede Usability and Immersion in the VR Environment

While participants had a positive experience with the VR stimuli, there were diverse comments about the usability and intuitiveness of the navigation interface/interaction paradigm. While several commented that they enjoyed the intuitive ability to kneel at ground level, to turn their body around physically, or to look around the environment in 360 degrees, several participants spoke about and were observed to have difficulty with the controllers. Participants mention that seeing the graphical interface of the controllers was distracting and impeded immersion, with a more natural body-based interface desired:

“*Seeing the controllers took me out. I looked down at them too much, relied too much. If I could not see them and just know how to use them, or maybe if there is another way for me to move and choose where I want to go without my hands, I’d really like that.*” P04, male
“*It was hard to be immersed when the controllers were needed. The interface was on screen so I kept seeing it, and it took me out a bit each time… body tracking, that would be more intuitive.*” P09, male

Another participant mentions the controllers didn’t feel easy to use, yet observational data demonstrates the participant appeared to get immersed in the environment by visibly flinching when virtual objects came near:

“*It was hard learning the controllers. I wanted to explore, but I had to keep looking at them to make sure I touched the right buttons.*” P08, female

Participants turned their head to look behind their peripheral vision an average of six times per experimental session; only one participant made a full physical circular turn with their body.

**To summarize**, the qualitative findings provide insight into the following research question (**RQ2**) “What effect(s) do the traits of aesthetic beauty/scale, familiarity, or personalization of the environment have on awe experiences?” For aesthetic beauty/scale, statements fitting into ‘Captured by the view/drawn to the phenomenon’ awe category were most numerous (9/16 participants). Given that notable awe-inspiring places share attributes of vastness, scale, and beauty (Keltner and Haidt, 2003; Shiota et al., 2007), the VR stimulus of the Earth fits this description well and is likely very captivating to observe. ‘Scale effects’ of perceptual vastness or largeness were mentioned by several, and while it isn’t a consensus awe category, the ‘small-self’, or diminished size phenomenon was mentioned in the scale effects context. ‘Aesthetic appreciation’ was mentioned by 5/16 participants, with comments reflecting their pleasure of seeing beautiful landscapes. With respect to ‘familiarity’, we see in the data that participants often chose familiar locations as their self-selected environment, such as hometowns and places family and friends reside. It is particularly interesting that hometowns are the most popular of all destinations participants visited, and it produced the most goose bump occurrences of all locations. Likely related to this finding is ‘personalization’, with most goose bumps occurring during the self-selected environment. We can determine from these findings that aesthetic beauty/scale positively influences awe, and familiarity/personalization of the environment leads to personal relevance that also positively influences awe.

Of all the categories, #3 “Connectedness (feeling connected with something without losing distinctness)” could potentially be the most interesting because “connectedness” statements indicated that participants might incite change because of their experience in VR. They used language such as “I should,” “I feel like I could, and “I think I need to,” which implies consideration of a possible real-world action once the VR experience is over. Finally, we see from the qualitative findings that usability challenges of the VR interface to navigate impeded some participants’ ability to become and stay immersed. From the statements, it is interesting that several participants intuitively knew their needs for a positive experience, as they identified potential benefits to a hands-free interface and no on-screen UI graphical elements. This is consistent with Slater et al. (1998) argument that natural, full body movement influences subjective presence, and in turn, immersion.

## Discussion

Overall our study showed that immersive interactive VR can indeed generate subjective experiences of awe and physiological goose bumps in many participants (**RQ1)**: four different interactive VR environments yielded an average awe rating of 79.7 (on a scale of 0–100). Our goose bump recorder instrument could indeed detect and record goose bumps, although we hope to automate goose bump detection in future design iterations. 43.8% of the participants experienced goose bumps, consistent with the ratio of goose bumps using stimuli in lab settings (Benedek and Kaernbach, 2011; Sumpf et al., 2015; Wassiliwizky et al., 2017). Also, awe ratings were positively correlated with the occurrence of goose bumps. Most goose bumps were experienced in the final self-selection VR environment. Personality traits yielded no clear correlation to awe ratings. While females had a higher incidence of goose bumps (66.7%, 4/6 females) than males (30%, 3/10 males), there was no difference between genders when it came to self-report ratings of awe. Our finding that females are more likely than males to experience goose bumps (**RQ3**) corroborates findings from two earlier studies that used only audio as stimulus (Panksepp, 1995; Benedek and Kaernbach, 2011), with a difference that our own study used audio and video as VR stimulus.

The observed positive correlation between ratings of humility and awe in our study is intriguing, since this same correlation was found by Gallagher et al. (2015) with participants that experienced awe also reporting higher humility. This may be related to the perception of a “*small self*” which is a shift in self-concept, the diminished size, that occurs during and after an awe-inspiring experience (Campos et al., 2013; Piff et al., 2015; Elk et al., 2016; Bai et al., 2017). This concept of small-self generated through awe is connected to feelings of humility, with humility often leading to improved feelings of social connectedness and well-being (Stellar et al., 2017a). As seen in the introspective interviews, we discovered participant statements that are illustrative of the small-self phenomenon, which may provide insight into the correlation between awe and humility ratings in the quantitative data.

In exploring (**RQ2**), we learn that aesthetic beauty/scale and familiarity/personalization of the environment positively influence awe. 12 categories of astronauts’ awe experiences based on Gallagher et al. (2015) were matched to participant interview statements. These ranged from impressions around the **aesthetics** being beautiful and awe-inspiring, to thoughts and feelings about **scale**. The scale comments involved both perceptual (‘the ocean is massive’) and conceptual impressions (‘it feels much greater than me’). In exploring the effect of **familiarity** and **personalization** (through self-selection of locations and agency) on awe, we found most goose bumps occurred during the final self-selected environment. Figure 14 displays the environments explored, and real-time occurrences of goose bumps. Many participants visited multiple places in the self-selection phase, since they were not limited to just one place. Iceland and Japan tied as the most visited with three visits each; respective participants stated they had never been to those places but always wondered what they would be like to travel to. Two participants opted to get an overview of the Earth from outer space as their self-selected option. Notably both those participants experienced goose bumps. Eleven participants used the self-selected environment phase to search for their hometown, place of birth, or ancestral home. These places weren’t always familiar to participants; in three cases, participants found the locations from their memory of photographs and descriptions. Of the eleven ‘home-traveling’ participants, three spoke of experiencing nostalgia. The elicitation of nostalgia has been correlated with maintenance and enhancement of a sense of meaning, or existential function (Routledge et al., 2011) and these are traits frequently seen in astronauts’ descriptions of the Overview Effect and awe (White, 2014). The nostalgic attributes demonstrated by some of the participants in our present study may reflect this previous research, with the feeling of curiosity to visit new places likely less indicative of awe. In terms of the selected height during navigation, five participants spent over half of their time orbiting space and taking an overview perspective of the Earth while trying to get from place to place, while the other eleven participants mainly stayed close to ground level, closer to airplane height.

Introspective data revealed that interactivity and allowing participants to navigate themselves had benefits, such as free movement and a feeling of agency, but also detriments, such as difficulty using controllers. This motivated us to create hands-free, leaning-based interfaces in our own design of awe-inspiring virtual environments (Quesnel et al., 2018). When looking at the participant’s demographic questionnaire details, several participants had experience with 3D games, but not VR systems. It is possible that the forward seated position of looking at a screen while playing games translates into a forward-facing habit in VR, with reliance on hand controllers to turn and navigate opposed to using the head and body. Indeed, the most experienced participants with VR tended to physically move their bodies the most on up/down, sideways, on rotation, and forward/backward. More exploration is needed in the use of interfaces that may be more universally intuitive or natural, such as leaning (Riecke et al., 2005; Kruijff et al., 2016; Kitson et al., 2017).

To summarize, these findings demonstrate that (1) interactive VR has an excellent capacity in eliciting awe, and if we regard goose bumps as a reliable indicator of awe, then familiar, self-selected environments are particularly effective; (2) physiologic goose bumps can be collected using a goose bump recorder instrument for non-intrusive, reliable indications of awe; (3) care must be taken to not impede awe and immersion or create distraction through appropriate design and intuitive, uncomplicated interaction interfaces; (4) While personality traits are not clearly correlated to awe ratings, goose bumps were experienced more frequently among females.

### Limitations and Future Directions

While our present study was able to explore the participants’ experience in considerable detail, the small sample size of 16 participants reduces the statistical power and opportunity to find significant effects. Additionally, we did not have an equal number of females to males, which is an improvement to be made in the next studies. This may have impacted the findings for (**RQ3**), where a higher percentage of females than males experienced goose bumps. Regardless, an equal proportion of males to females in addition to a larger sample size may yield more conclusive results. Based on pilot testing results as described in subsection “Virtual Environment Locations and Order,” we chose a fixed instead of counterbalanced or Latin Square order of the four scenes. Thus, order was a potential confound, and the finding that self-selected environment (also the last environment) provided the most goose bumps might change if order was balanced, which future research should investigate. Nevertheless, the qualitative data indicated that the self-selected environments had personal and emotional relevance for many participants, which seems less likely to be affected by order effects.

A limitation may be the use of one researcher to conduct interviews and analyze the qualitative open-ended, semi-structured interviews. To guard against subjective idiosyncrasies and promote rigor and justification of claims, it could be advantageous to utilize two or more researchers for the qualitative analysis. Since the categories of awe were provided via Gallagher et al. (2015, p 29) with comprehensive examples of category statements provided in text, we feel confident that statements were classified to the best of the researcher’s ability and acknowledge that the categories may in fact evolve as our understanding of awe increases. Gallagher et al. (2015) framework is thoughtful and clearly strives for validity, and to understand the complexity of the framework it took considerable time for our researcher to apply and check the accuracy of the framework. Training of more researchers in understanding the process could build more rigor.

Through semi-structured interviews, a predominant usability concern voiced by participants was with the operability of the VR hand controllers. Ten of the sixteen participants were observed to struggle with the controllers during the experience (seen in interactions where the controllers led to participants getting “stuck” in place while flying through the VR environment through incorrect button pushing, or gesturing). Researchers also noticed participants had high reliance on the controllers in navigating the virtual environment and did not use their head position or body position changes to look around the environment. With their gaze largely directed at the on-screen controls, and not constantly at the environment itself means the controllers themselves may have broken the level of presence and immersion, illustrated by participant comments like “it took me out.” The lack of physical body turning (as in, rotating the body to change orientation) from participants should also be taken into discussion, since this is a frequent and debatable point in creation of cinematic VR content; where and how do we direct the gaze, and prompt emotional moments? Covert and overt orienting are often embedded within a VR narrative to direct attention, with immersive audio (Jerald, 2016). Given the Google Earth VR environment is open and exploratory, it would be beneficial to explore directed narratives as stimulus for covert and overt orienting. Provided that awe contains two key features, “vastness” and the “need for accommodation,” Cohen et al. (2010) investigated differences between narratives on profound beauty (as aesthetics) and spiritual transformation narratives (as natural phenomenon, relationships, sacredness) and discovered that transformative narratives seem to produce long-lasting change over aesthetic narratives. For future directions with VR content on eliciting awe and transformative emotions, guided narrative could be compared with an open-world environment.

Usability concerns and user interface (UI) distraction points to limitations with a commercially available application (Google Earth VR) not purpose-built to induce awe. Remarkably, while not an intentional design feature, elicitation of the Overview Effect through Google Earth VR has been experienced by some (Podwal et al., 2016). This points to how VR as a nascent medium can produce surprising, unintended positive effects with opportunities to explore dedicated design for awe and the Overview Effect.

Interestingly, two participants described the passive 5-min Color Tour at the start of the experiment to be their favorite environment. This introduces the potential for limited-interactivity VR to potentially be more effective in allowing concentration on aesthetics and the experience of ‘being there’. Once hand controllers are used, focus may shift to completing tasks (flying, or searching) rather than simply “being there.” It is worth investigating how navigation interfaces with different modes of interaction impact awe and embodiment. Intuitive navigation interfaces like leaning and gestural motion tracking should be explored; our recent study using leaning-based interaction in a VR environment designed to elicit awe and wonder suggests that providing embodied navigation and eliminating hand controllers may reduce breaks in presence (Quesnel et al., 2018). In future studies, avoidance of interactions that may prevent awe from occurring through a loss of presence or concentration could provide evidence of new typologies of virtually induced awe.

In exploring a potential relationship between the small-self, humility and awe-inspiring experiences, a formal measure could be used such as the Perceived Self-Size Scale (Bai et al., 2017). Likewise, the relationship between experiencing awe and feeling a desire to socially connect was illustrated in some of the qualitative themes of this study, and a measure such as the Inclusion of the Other in the Self Scale (Aron et al., 1992) to collect data on social connectivity may be valuable. With these scales, it may be possible to investigate if the VR environment and interaction with it can impact a participant’s perception of themselves and connection to others. As immersion and presence might be important factors enabling awe experiences, presence and immersion questionnaires could be used such as the Presence Questionnaire (PQ) by Witmer and Singer (1998) or Slater-Usoh-Steed (SUS) questionnaire by Usoh et al. (2000). A possible circular effect exists of emotions in the VR environment influencing presence levels that in turn influence emotions felt (Riva et al., 2007; Diemer et al., 2015), so data on presence correlating with emotions could be valuable.

Through additional physiological data, we may better understand patterns in the experience of awe. Biosensors, like SCR and Electrocardiogram (ECG) worn in addition to the goose bump instrument may provide real-time data demonstrating a relationship between processes of the autonomous nervous system. Through triangulation of data, it may be possible to differentiate between awe and frequently concurring emotions, like fear, and recognize emotions in order to provide biofeedback into a VR system (Quesnel et al., 2017). Other studies exploring markers of health and awe demonstrated that a tendency to feel awe predicts lower levels of pro-inflammatory cytokines compared to other emotions (Stellar et al., 2015). Inflammation is attributed to negative cardiac health and numerous auto-immune conditions, so investigating if awe in VR can lead to similar findings for improved wellness would be compelling.

## Conclusion

Despite the limitations on the controller-based navigation interface and common personality tests unable to predict awe, results from this study demonstrate that immersive VR can be effective in eliciting awe, measured through physiological and self-report data. With immersive VR increasingly recognized for its potential to evoke shifts in a user’s beliefs and values, future experiments could use high-quality multisensory VR stimuli to maximize emotions and engagement. Findings encourage exploration of VR stimuli and interaction paradigms for awe experiences and self-transcendent emotions that potentially lead to lasting, positive well-being and social implications. For further insight into awe experience, we will conduct future studies with custom-designed VR content and interfaces as a Positive Technology for awe elicitation, and will complement the goose bumps measure with additional physiological instruments.

## Author Contributions

DQ and BR conceived the main idea of the article. DQ collected data, conducted statistical and qualitative analysis, and wrote the first draft of the manuscript. BR provided guidance on the data analysis and rhetoric, and supervised the entire work. All authors contributed to the manuscript, read, revised, and approved the final version.

## Conflict of Interest Statement

The authors declare that the research was conducted in the absence of any commercial or financial relationships that could be construed as a potential conflict of interest.
